# The natural history of West Nile virus infection presenting with West Nile virus meningoencephalitis in a man with a prolonged illness: a case report

**DOI:** 10.1186/1752-1947-5-204

**Published:** 2011-05-25

**Authors:** Shraddha Mainali, Mansoor Afshani, James B Wood, Michael C Levin

**Affiliations:** 1 Department of Neurology, University of Tennessee Health Science Center, Memphis, TN 38163, USA; 2 Neurology Service, Veterans Administration Medical Center, Memphis, TN, USA; 3 Radiology Services, Veterans Administration Medical Center, Memphis, TN, USA

## Abstract

**Introduction:**

Estimates indicate that West Nile virus infects approximately one and a half million people in the United States of America. Up to 1% may develop West Nile virus neuroinvasive disease, in which infected patients develop any combination of meningitis, encephalitis, or acute paralysis.

**Case presentation:**

A 56-year-old African-American man presented to our hospital with headache, restlessness, fever, myalgias, decreased appetite, and progressive confusion. A cerebrospinal fluid examination showed mild leukocytosis and an elevated protein level. Testing for routine infections was negative. Brain T2-weighted magnetic resonance imaging scans showed marked enlargement of caudate nuclei and increased intensity within the basal ganglia and thalami. A West Nile virus titer was positive, and serial brain magnetic resonance imaging scans showed resolving abnormalities that paralleled his neurological examination.

**Conclusion:**

This report is unusual as it portrays the natural history and long-term consequences of West Nile virus meningoencephalitis diagnosed on the basis of serial brain images.

## Introduction

West Nile virus (WNV) is an arthropod-borne flavivirus transmitted to humans by the bite of an infected mosquito [1,2]. The flavivirus belongs to the Japanese encephalitis virus antigenic complex, which was first isolated from a 37-year-old woman living in the West Nile District of Uganda in 1937 [1]. Sixty-two years later, in the summer of 1999, the virus was first identified in the United States of America, where it appeared during an outbreak of naturally acquired meningitis and encephalitis in the New York City area [3].

WNV infection typically peaks in late July through early September [4]. The disease usually presents with three clinical syndromes: asymptomatic infection, mild febrile syndrome (WNV fever), and neuroinvasive disease. The majority of people infected are asymptomatic [4,5]. Approximately 20% of people who are infected with WNV develop WNV fever, which presents as a flulike illness (headache, malaise, myalgias, and lymphadenopathy) and a non-specific maculopapular rash involving the neck, trunk, arms, and legs [5]. About 1% of WNV infections result in WNV neuroinvasive disease (WNND), defined by evidence of WNV infection with any combination of meningitis, encephalitis, or acute flaccid paralysis or poliolike syndrome [5].

In 1999, the Centers for Disease Control and Prevention (CDC) reported a total of 62 WNV infections, of which 59 presented with WNND [4]. There were seven fatalities. The number of WNND cases peaked in 2002. That year there were 2946 WNND cases and 284 deaths [4]. The latest data from the CDC show a total of 21 cases of WNND with two deaths in 2010 [6]. It has been reported that the incidence of WNND ranges between 1:140 and 1:256 among people infected with WNV [4]. By extrapolation, the virus has infected more than one and a half million people in the United States of America, and the long-term disability following WNND is just beginning to be appreciated [4,7,8]. We present a case of one of the survivors of WNND. The significance of this case is that serial brain magnetic resonance imaging (MRI) scans were obtained which correlated with the clinical course of the disease, hence supporting the use of brain MRI in rendering a preliminary diagnosis and following the progression of WNND.

## Case presentation

A 56-year-old African-American man with a history of hypertension and chronic hepatitis C virus infection presented to the emergency room with a three-day history of flulike symptoms, including fever, myalgias, headache, and decreased appetite. His vital signs were blood pressure 204/104 mmHg, pulse 90 beats/minute, respiratory rate 14 breaths/minute, and peak temperature 105.2°F. His physical examination was notable for restlessness and confusion. There was neck rigidity. Papilledema was not present. He could not follow commands, and mild right hemiparesis was noted. Computed tomography of the brain showed a subtle, low-density signal of the caudate nuclei bilaterally (not shown). His serum laboratory values were normal except for mild leukocytosis, with a total white blood cell count of 10,400/mm^3 ^comprising 82% neutrophils, 9% monocytes, and 8% lymphocytes. His cerebrospinal fluid (CSF) examination showed white blood cell count of 29/mm^3^, comprising 81% lymphocytes, 15% neutrophils, 4% monocytes, 87 mg/dl protein, and 63 mg/dl glucose. We performed CSF Gram staining, acid-fast bacilli staining, a meningitis screen (*Neisseria meningitidis*, group B Streptococcus, *Streptococcus pneumoniae*, and *Haemophilus influenzae *type b), a cryptococcal antigen test, and a Venereal Disease Research Laboratory test, all of which were negative.

**Figure 1:**
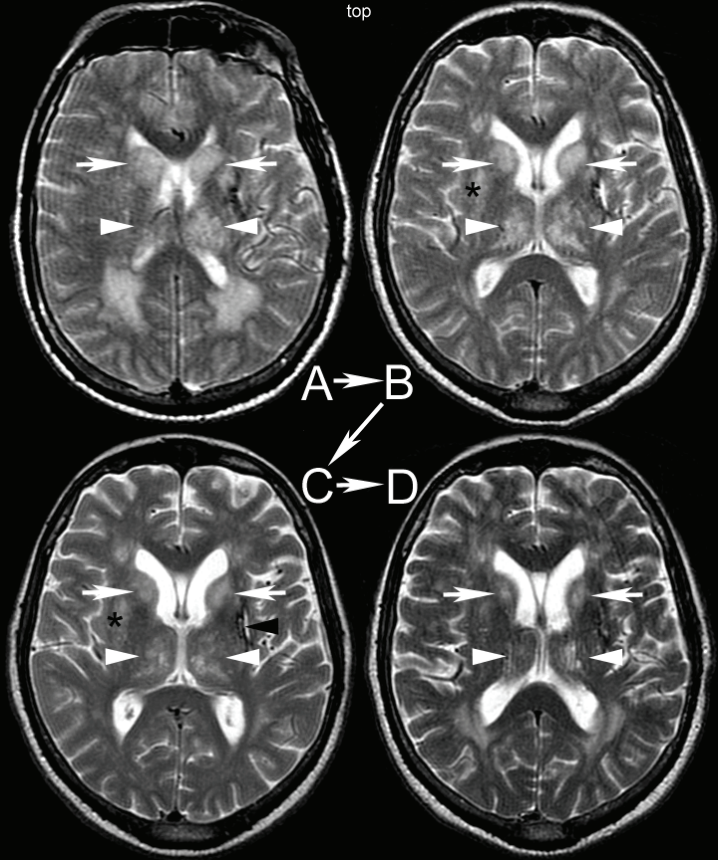
**Serial brain magnetic resonance imaging (MRI) scans obtained during the course of the patient's illness**. **(A) **Day 2: T2-weighted MRI scan demonstrates markedly enlarged caudate nuclei (arrows) and increased intensity in the thalami (arrowheads). **(B) **Eleven days after his initial presentation the abnormalities were resolving (arrows and arrowheads). Of note, the lenticular nucleus was involved (asterisk). **(C) **Three weeks and **(D) **two months after his initial presentation the abnormalities continued to improve, but had not resolved completely (arrows, arrowheads). Incidental hypertensive hemorrhage was present (black arrowhead in Figure 1C).

He was admitted to the intensive care unit in a stupor. He was treated with broad-spectrum antibiotics (vancomycin, ceftriaxone, ampicillin, and tetracycline) and intravenous acyclovir. Laboratory evaluations for herpes simplex virus (HSV)-1 and HSV-2, tuberculosis (TB), cytomegalovirus (CMV), and WNV were sent. On day two, an electroencephalogram showed diffuse slowing without seizure activity. A brain MRI performed on the same day demonstrated markedly enlarged caudate nuclei and increased intensity of caudate, lenticular nuclei and the thalamus on T2-weighted images (Figure 1A). T1-weighted images showed a subtle, low-intensity signal within the lesions, and the lesions did not enhance following gadolinium infusion (not shown). He continued to have fever and confusion with a waxing and waning mental status. Polymerase chain reaction studies of his CSF for HSV, TB, and CMV were negative. His blood, urine, and CSF cultures were negative. By day seven, his mental status had improved to the point that he was oriented to himself and could state his age. From hospital days eight to 16, he showed slow improvement with phases of intermittent confusion. On day 13, a brain MRI scan (Figure 1B) showed decreased edema in the basal ganglia and thalamus as compared to the prior MRI scans.

On day 19, he was fully oriented, and his mental status appeared to have returned to baseline. His WNV immunoglobulin M (IgM) titer at that time was positive (acute and convalescent phase). A brain MRI scan (Figure 1C) obtained on day 21 revealed resolving inflammatory changes in the basal ganglia and thalamus. Six weeks later he was fully oriented to time, place, and person and did not articulate any complaints. Another brain MRI scan (Figure 1D) showed resolving basal ganglia and thalamus edema with persistent hyperintense changes in both structures. Approximately one year later he was diagnosed with depression. Four years later formal neuropsychological and neurological evaluations showed evidence of difficulties with motor and mental processing speed and residual, mild right hemiparesis.

## Discussion

This case exemplifies a form of WNND presenting as WNV meningoencephalitis. The most reliable diagnostic modality for WNV infection is the detection of serum IgM antibody to WNV in his serum collected within eight to 14 days of presentation or CSF collected within eight days of the onset of illness using IgM antibody capture enzyme-linked immunosorbent assay [5]. Considering the relatively long interval between the sample collection and reporting of the definitive test, alternative methods of diagnosis would be helpful in prompt management of the disease. In our patient, we found that the brain MRI findings (bilateral edema and hyperintensity of the basal ganglia and thalami) were helpful in this clinical setting to establish a preliminary diagnosis of WNND. Further, since clinical improvement correlated with resolving changes on serial brain MRI scans, his prognosis could also be assessed. Critically, the brain MRI scan did not return to normal, nor did he, indicative of the long-lasting effects of WNND, which include residual psychological and psychiatric disease [7-9]. Consistent with these observations, our patient developed cognitive dysfunction with depression and is still undergoing outpatient psychological treatment. Of note, these MRI findings are not specific to WNV encephalitis. Other viral illnesses such as Japanese encephalitis virus and St Louis encephalitis virus can show similar findings; thus brain MRI cannot be the sole diagnostic modality for detecting WNND [2]. For example, recent data indicate that chorioretinitis may be a marker of WNND [10]. However, if other risk factors such as geographic location (WNV predominant locations), history of exposure to mosquitoes, and the time of the year are considered, brain MRI can be useful for establishing an early diagnosis and treatment plan while the definitive test is pending.

## Conclusion

Characteristic patterns of serial brain MRI scans in patients with WNND can provide an early clinical clue as to the diagnosis and prognosis while awaiting definitive laboratory testing.

## Abbreviations

CDC: Centers for Disease Control and Prevention; CMV: cytomegalovirus; CSF: cerebrospinal fluid; HSV: herpes simplex virus; MRI: magnetic resonance imaging; TB: tuberculosis; WNND: West Nile virus neuroinvasive disease; WNV: West Nile virus.

## Consent

Written informed consent was obtained from the patient for publication of this case report and any accompanying images. A copy of the written consent is available for review by the Editor-in-Chief of this journal.

## Competing interests

The authors declare that they have no competing interests.

## Authors' contributions

SM and MA analyzed and interpreted the patient data regarding the clinical presentation and were major contributors in writing the manuscript. JW performed and interpreted the MRI data. MCL reviewed all of the data and made major contributions to the writing and editing of the manuscript. All authors read and approved the final manuscript.
